# Complete Genome Sequence of Strain PM004, a Novel Cultured Member of the Human Oral Microbiome from the Candidate Phylum *Saccharibacteria* (TM7)

**DOI:** 10.1128/MRA.01159-19

**Published:** 2019-10-17

**Authors:** Pallavi P. Murugkar, Andrew J. Collins, Floyd E. Dewhirst

**Affiliations:** a The Forsyth Institute, Cambridge, Massachusetts, USA; b Department of Oral Medicine, Infection and Immunity, Harvard School of Dental Medicine, Boston, Massachusetts, USA; Indiana University, Bloomington

## Abstract

Strain PM004 is a cultured representative of human microbial taxon 955, a bacterium from the phylum *Saccharibacteria*. It is an obligate parasite with a genome of <0.9 Mb and can be grown in coculture with its host, Pseudopropionibacterium propionicum. The complete genome sequence is presented here.

## ANNOUNCEMENT

**FIG 1:**
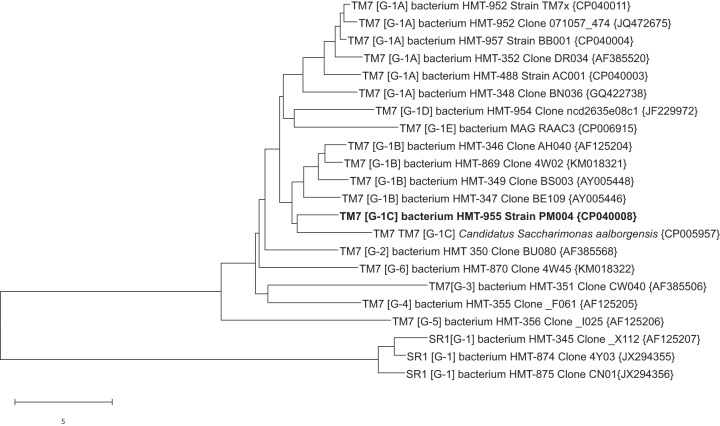
Neighbor-joining tree (10) for *Saccharibacteria* (TM7) bacterium HMT 955 strain PM004. The tree was produced from aligned full-length 16S rRNA sequences (∼1,450 bp) using MEGA X (11). The designations in square brackets are the TM7 class level groups as described in reference 12. GenBank accession numbers for 16S rRNA or genome sequences are given in curly brackets. The scale bar shows a 5% difference in sequence similarity. For trees showing broader TM7 diversity, see references [Bibr B12][Bibr B13][Bibr B14].

The human oral cavity contains many previously uncultivated bacteria, including members of the candidate phyla radiation (CPR) (1, 2). Oral isolate TM7x (3, 4) was the first CPR bacterium cultured. This member of the candidate phylum *Saccharibacteria* was isolated in coculture with Actinomyces odontolyticus strain XH001 and is thought to be an obligate parasitic epibiont (5, 6). Strain TM7x is a member of human microbiome taxon (HMT) 952 in the Human Oral Microbial Database (7, 8). The human oral *Saccharibacteria* strain PM004 was isolated in coculture with Pseudopropionibacterium propionicum strain F0700. HMT 955 strain PM004 differs sufficiently from HMT 952 strain TM7x by both 16S rRNA (93.7% identity) and genome comparisons to represent a novel genus and species. A phylogenetic tree for human oral and important environmental *Saccharibacteria* is presented in Fig. 1. Strain PM004 was isolated in Cambridge, MA, from the subgingival plaque of a 47-year-old Asian male smoker with periodontitis. The sample was dispersed and filtered through a 0.2-μm filter to remove all but ultrasmall bacteria and was added to broth cultures of *P. propionicum.* The novel *Saccharibacteria* bacterium was recovered growing in coculture with *P. propionicum.*

DNA for genome sequencing was isolated from a 200-ml coculture of strain PM004 and *P. propionicum* in Trypticase soy broth with 1% yeast extract. The cocultured PM004 cells were filtered through a 0.45-μm filter and pelleted by centrifugation. DNA was isolated from cells by bead beating and through the use of a MasterPure DNA isolation kit (Lucigen). Library preparation and DNA sequencing were performed at the Johns Hopkins Deep Sequencing and Microarray Core. Genomic DNA was sheared to 10 to 20 kb using a Covaris g-TUBE and purified using AMPure XP beads (Agencourt Bioscience). Size selection and further cleanup were performed using BluePippin (Sage Science). The library was sequenced on a PacBio RS II instrument on one single-molecule real-time (SMRT) cell per library. Default parameters were used for all software. The reads were assembled using the SMRT Analysis software version 2.3.0 HGAP3 pipeline. Methylation motifs were detected using the SMRT Analysis software version 2.3.0 Base_Modification_and_Motif_ Analysis pipeline. Genes were annotated using the NCBI Prokaryotic Genome Annotation Pipeline using the best-placed reference protein set (GeneMarkS-2+ v4.8) (9).

There were 80,150 postfilter reads covering 1,166,362,784 sequenced bases. The mean read length was 14,552 bases, and the *N*_50_ read length was 21,336 bases. The reads were assembled into 21 contigs. The genome of HMT 955 strain PM004 was present in a single contig of 864,547 bp with average reference coverage of 918×. It was circularized to 842,202 by removing a duplicated 22-kbp segment at the ends of the initial contig. Twenty other contigs of less than 54 kbp and 40× coverage were identified as low-level contamination of host bacteria. The GC content of the DNA was 46.8%. Genome annotation identified a total 875 genes, of which 825 were predicted to be coding sequences (CDSs), 50 were RNAs, and 10 were pseudogenes.

### Data availability.

The genome sequence was deposited at GenBank under accession number CP040008 and SRA accession number SRR9734868. Base modification files were submitted with the GenBank accession.
